# Tannic acid attenuates glyphosate-based herbicide-induced dopaminergic neurotoxicity associated with motor dysfunction in mice

**DOI:** 10.3389/fnbeh.2026.1785573

**Published:** 2026-04-17

**Authors:** Patrick Oluwole Abolarin, Bamidele Victor Owoyele

**Affiliations:** 1 Department of Physiology, Faculty of Basic Medical Sciences, University of Ilorin, Ilorin, Nigeria; 2 Department of Physiology, School of Basic Medical Sciences, Babcock University, Ilishan-Remo, Nigeria

**Keywords:** antioxidant enzymes, glyphosate, neuroinflammation, Parkinson’s disease, tannic acid

## Abstract

**Introduction:**

Glyphosate, a widely used herbicide, has gained attention due to its potential link to neurobehavioral and dopaminergic dysfunctions. Data on interventions against glyphosate-induced neurotoxicity are limited. Hence, the neuroprotective role of tannic acid (TA), a polyphenolic anti-inflammatory and antioxidant agent, was investigated in mice chronically exposed to glyphosate-based herbicide (GBH).

**Methods:**

Male Swiss mice were randomly allocated into six groups (*n* = 8) and received daily oral gavages of specific solutions that were prepared daily: Control (distilled water 1 mL/kg body weight), GBH (500 mg/kg body weight), Pre-TA + GBH (TA 50 mg/kg body weight, pre-treated, then GBH-exposed), TA + GBH (TA 50 mg/kg body weight and GBH-co-treatment), Pre-AA + GBH [ascorbic acid (AA) 100 mg/ kg body weight, pre-treated, then GBH-exposed], and AA + GBH (AA 100 mg/ kg body weight and GBH-co-treatment). Motor function tests, biochemical, and histological analyses of the midbrain were performed 6 weeks post-treatment.

**Results:**

TA significantly inhibited GBH-induced motor dysfunction. As compared to the GBH group, TA treatments significantly (p < 0.0001) decreased midbrain malondialdehyde (MDA), TNF-α, IL-1β, and IL-6 levels. TA treatments increased significantly (*p* < 0.0001) the concentrations of dopamine, the activities of catalase (CAT), glutathione peroxidase (GPx), and superoxide dismutase (SOD) enzymes in the midbrain relative to the GBH group. These effects were similar to those of the control and AA-treated mice. Conclusively, TA ameliorated GBH-induced motor dysfunction in mice and attenuated associated midbrain oxidative stress, inflammatory responses, and dopaminergic alterations.

**Discussion:**

These findings are suggestive of the neuroprotective effects of TA against environmental toxicant-induced neurotoxicity.

## Introduction

The widespread use of pesticides in residential settings, landscaping, and agriculture has been associated with potential health risks in humans and increased risk of environmental contamination through various xenobiotics (Dereumeaux et al., 2020). Glyphosate-based herbicides (GBHs) are among the most widely used herbicidal formulations globally due to their weed control potency in the agricultural and horticultural domains. Glyphosate, the active ingredient in GBHs, including Roundup, performs its herbicidal action through inhibition of the shikimate pathway in plants. Although this pathway is absent in humans and animals, emerging evidence suggests that chronic exposure to GBH may exert deleterious effects beyond its herbicidal effect, including potential neurotoxic effects on the nervous system (Klátyik et al., 2025; Mazuryk et al., 2024). Epidemiological studies have demonstrated that human GBH exposure is rising, and the current safety standards for GBH are outdated and may fail to protect the environment and public health in general (Mazuryk et al., 2024).

Reports from experimental studies have demonstrated that chronic exposure to GBH can stimulate the generation of reactive oxygen species, mitochondrial dysfunction, and neuroinflammatory responses in the central nervous system (Winstone et al., 2022; Madani and Carpenter, 2022). Indeed, these mechanisms are canonical contributors to neuronal dyshomeostasis and behavioral impairments. Specifically, several animal reports have shown that chronic glyphosate exposure may cause alteration of dopaminergic neurotransmission, resulting in mood alteration, memory impairments, and particularly motor abnormalities, suggesting the vulnerability of the nigrostriatal system to glyphosate-induced neurotoxicity. In agreement with these observations, Ait Bali et al. (2017) reported hypokinesia associated with anxiety and depression-like behaviors in mice exposed to glyphosate both sub-chronically and chronically, although there was no alteration in locomotor function with respect to acute glyphosate exposure. In the same vein, Hernández-Plata et al. (2015) demonstrated that intraperitoneal administrations of glyphosate induced hypoactivity in adult male rats.

Because the degeneration of the nigrostriatal dopaminergic system plays an integral role in the regulation of motor function, environmental factors capable of disrupting the dopaminergic signaling have received significant attention in neurodegenerative research. The development and progression of many neurological disorders, particularly those involving dopaminergic dysfunction, including Parkinson’s disease (PD), have been associated with environmental factors (Li et al., 2021). PD, a progressive neurodegenerative condition characterized by progressive motor dysfunction, cognitive impairment, and a spectrum of non-motor symptoms (Abolarin et al., 2022), results from the degeneration of dopaminergic neurons in the SNpc. Although the exact etiology of PD remains elusive and multifactorial, convincing evidence suggests that a complex interplay between genetic susceptibility and environmental factor exposures, including certain pesticides and herbicides, may contribute to dopaminergic neurodegeneration (Abolarin et al., 2024).

Several mechanisms have been proposed to explain how exposure to GBH induces neuronal injury. One important mechanism involves oxidative stress, where GBH exposure potentiates the production of reactive oxygen species (ROS), resulting in neuronal injury and death (Martínez et al., 2020). It has also been reported that glyphosate disrupts mitochondrial function, thereby impairing energy biogenesis in the neural cells that are particularly susceptible to oxidative damage, including dopaminergic cells in the SNpc (Moser et al., 2022). In the same vein, studies have shown that glyphosate is a promoter of neuroinflammation either in the peripheral nervous system or in the central nervous system by activating microglia, leading to the release of proinflammatory cytokines that aggravate neuronal loss (Izumi et al., 2024). Furthermore, glyphosate has been reported to interfere with tyrosine hydroxylase (TH), a rate-limiting enzyme in the monoamine pathway, thereby reducing dopamine and neurotransmission (Madani and Carpenter, 2022). Collectively, this climaxes the progression of dopaminergic neuron degeneration in the SNpc, leading to motor dysfunction. Therefore, investigating environmental neurotoxicants that may alter the dopaminergic nigrostriatal axis is germane for underpinning mechanisms that could contribute to toxin-induced motor dysfunction. Understanding these mechanisms would assist in identifying potential neuroprotective agents, including TA, a polyphenolic compound.

Tannic acid is a naturally occurring polyphenolic compound derived from fruits, nuts, and plant-derived beverages. TA has gained significant attention for its potent antioxidant, anti-inflammatory (Jing et al., 2022), and metal-chelating properties (Chariyarangsitham et al., 2021). Importantly, experimental reports have shown the capacity of TA to modulate oxidative stress signaling pathways, inhibit activation of nuclear factor-kappa B (NF-κB), and mitigate the production of pro-inflammatory cytokines (Jing et al., 2022). These properties suggest the neuroprotective potential of TA against neurotoxicant-induced neuronal injury, importantly in conditions related to oxidative stress and neuroinflammation. GBH exposure is associated with oxidative stress, neuroinflammation, and mitochondrial dysfunction, mechanisms that are mostly implicated in dopaminergic neurotoxicity and associated motor dysfunction. Zhang et al. (2023) reported in an *in vitro* study the capacity of tannins to reduce glyphosate-induced neuroinflammation, oxidative impairment, and apoptosis. Therefore, the present study investigated whether TA could attenuate GBH-induced neurochemical alterations and associated motor dysfunction in mice.

## Materials and methods

The Male Swiss Albino mice (weighing 25–30 g) used in the study were procured from the animal house of the Central Research Laboratory (CRL), University of Ilorin, Nigeria. Following the procurement of the mice, they were allowed to acclimatize to the laboratory for 7 days before experimentation. Mice were housed in plastic cages (8 mice/cage, 45 cm × 20 cm × 15 cm) with sawdust as bedding. The mice were kept in a hygienic environment with temperature conditions sustained at 24 ± 2 °C and a light/dark cycle of 12 h/12. Animals were *ad libitum* given commercially available rodent pellets (Topfeeds Rodent Pellets), manufactured by Premier Feed Mills Co. Ltd., a subsidiary of Flour Mills of Nigeria Plc, and replenished daily with distilled water (CRL, University of Ilorin, Nigeria), which was not subjected to any further treatment throughout the experiment.

### Chemicals

Mice were exposed to glyphosate using the known GBH, Roundup Turbo herbicide (Monsanto Co., St. Louis, MO; Monsanto of Brazil Ltda, São Paulo, Brazil) that contained glyphosate as the active ingredient. The formulation of Roundup (molecular formula C_6_H_17_N_2_O_5_P, molecular weight of 228.183 g/mol, melting point 200 °C, and density 1.218 g/cm^3^) contains glyphosate at 450 g/L and isopropylamine salt at 648 g/L. The TA (CAS No: 1401-55-4) and AA (CAS No: 50–81-7) used were procured from Sigma Chemical Co. (St. Luis, MO, United States) and prepared in distilled water.

### Experimental design

In total, 48 male Swiss Albino mice were divided randomly into 6 groups, with each group containing 8 mice. The groups received either distilled water only, GBH, TA or AA via oral gavage using a gavage needle as follows: Control (distilled water, 1 mL/kg b.w.), GBH (500 mg/kg b.w.), Pre-TA + GBH (7-day Pre-treatment with TA, 50 mg/kg b.w.) followed by concurrent GBH exposure, TA + GBH (co-treatment with TA, 50 mg/kg b.w. and GBH), Pre-AA + GBH (7-day Pre-treatment with AA, 100 mg/kg b.w. followed by concurrent GBH exposure), and AA + GBH (co-treatment with AA, 100 mg/kg b.w. and GBH). The GBH group was given glyphosate in liquid commercial form at a concentration of 500 mg/kg. Administration of drugs began when mice were 8 weeks old, an age corresponding to the early adult stage in mice. Ascorbic acid (AA) used in this study served as a reference antioxidant on account of its canonical anti-inflammatory, antioxidant, and neuroprotective properties. Hence, in this study, AA serves as a positive control to benchmark the potential antioxidant and neuroprotective effects of TA, providing the avenue to compare the effects of TA with a well-known antioxidant agent.

The glyphosate dosage (500 mg/kg b.w.) was selected on the basis of no observable adverse effect level (NOAEL) for maternal and developmental toxicity in rabbits, which served as the reference dose for the definition of tolerable daily consumption for glyphosate. Moreover, EPA U (1993) reported the NOAEL of 500 mg/kg/day for mice in sub-chronic and chronic glyphosate exposures. Although this dose is higher than the concentration that the general population is typically exposed to Tarazona et al. (2017), it is in agreement with previous toxicological reports that investigated glyphosate’s mechanism of action. In agreement with this, Ford et al. (2017) reported that several toxicological studies with pesticides are performed at high concentrations so as to reveal the potential mechanism of action of the chemical. Essentially, the dose of glyphosate used in this study allows demonstration of measurable neurochemical and oxidative stress alterations while remaining within sub-lethal exposure ranges in rodents. The dosage of TA was selected on the basis of previous reports demonstrating antioxidant and neuroprotective effects in rodent models without evidence of systemic toxicity (Bona et al., 2022; Azimullah et al., 2023; Soyocak et al., 2019), while that of AA was based on previous reports as well (Iwata et al., 2010; Ekanem et al., 2020).

**Figure 1:**
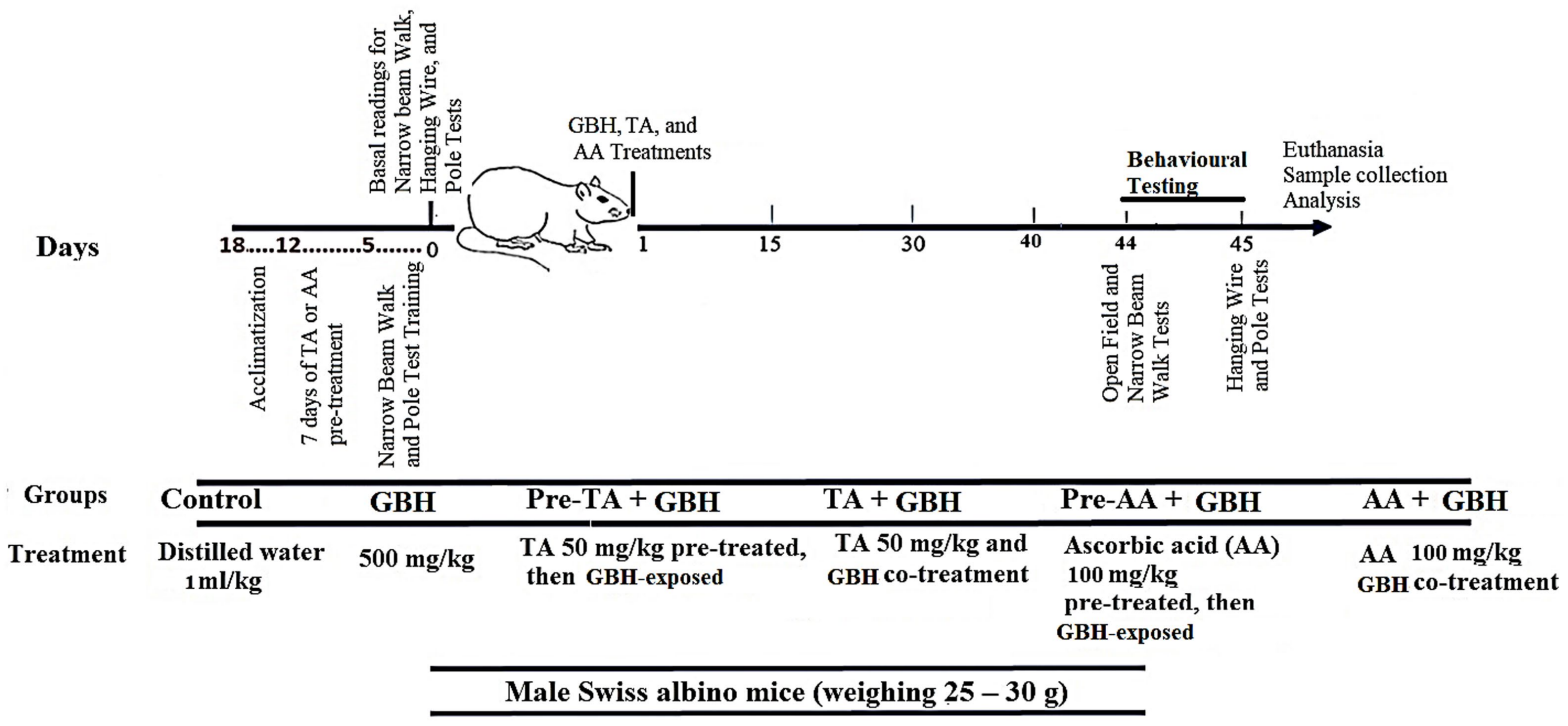
Experimental design.

Following acclimatization, mice were trained for 5 days on a gradual process of familiarization and encouraging successful traversals on the narrow beam and pole tests. Subsequently, baseline line readings were taken for the narrow-beam walk, pole, and hanging wire tests to assess motor coordination and balance of the mice before treatment. Mice were chronically exposed to GBH for 45 days. The 45-day period of exposure was chosen to model chronic exposure, which is often used in toxicological studies to investigate cumulative effects and progressive oxidative stress after continuous exposure to pesticides. On the 44th day of the experiment, between 09:00 and 13:00 h, Open Field and Narrow Beam Walk tests were assessed, while on the 45th day, Hanging Wire and Pole Tests were carried out to re-evaluate the mice’s motor function (Figure 1). For the two tests conducted per day, a 30-min inter-test interval (ITI) was ensured for stress reduction and minimization of carryover effects. Furthermore, this order of neurobehavioral testing was kept consistent for all the mice to ascertain comparability. All the mice (*n* = 8) went through behavioral. Following the behavioral tests, the animals were randomly assigned to respective analyses: five (*n* = 5) animals were used for biochemical analysis, while the remaining three (*n* = 3) were also euthanized and processed for histological evaluation of brain tissue. Rats were continuously monitored on a daily basis for signs of distress, abnormal behavior, or adverse reactions to drug administrations. There were no mortalities or severe treatment-associated complications observed throughout the period of the study.

### Open field test (OFT)

The open field test was chosen to evaluate the general locomotor function and exploratory activity of the mice. Each mouse was positioned at the center of a novel box made of wood, measuring 40_40_40 cm (Wilson et al., 1976), and with black wood flooring. Activities of the mice were measured for 5 min and monitored through video recording using a video camera equipped with ANY-maze (version 7.3, Stoelting CO, United States) tracking program. After each trial, the maze was cleaned with 70% ethanol. A lamp (75 W), giving a light intensity of about 30–40 lux at the center of the arena. However, the lamp was not placed directly facing the open field apparatus to prevent the generation of anxiety, but positioned in a porthole diffusing light and situated at about 250 cm from the test arena, allowing the center of the test arena to be seen under a dim light. Locomotor (total distance covered and speed) and rearing (given as full vertical posture on the mouse hind limbs, either with forelimbs touching the wall or unsupported) activities of the mice were evaluated after the animals were placed individually into the open field maze.

### Narrow beam walk test

A narrow beam maintained at the dimensions of (L 100 cm × W 1 cm) and supported by two wooden towers was used. The narrow beam was hung at a height of 1 m above the floor. A box with a target hole that permits mice to enter from the beam into the box was fixed to one end of the beam. Animals were trained to cross the beam. The motor coordination was evaluated by measuring the traversal time of the animals to cross the beam. A maximum cut-off time of 120 s was set for mice to traverse the narrow beam. A mouse that fails to reach the target box within the 120-s maximum cut-off time is assigned a traversal latency score of 120 s for statistical analysis.

### Hanging wire test

The hanging wire test was included to investigate neuromuscular strength and endurance, parameters regularly implicated in neurotoxicant-induced motor impairment. Evaluation of endurance and gripping abilities or strength was performed through the hanging wire test as previously described by Rakhunde et al. (2014). Mice were gently held and hung to a string positioned 45 cm above a foam sheet floor and extended across two poles using their forelimb. The foam sheet was placed underneath the string to avoid potential injury in case of a fall. The fall threshold of the mice was calculated in seconds (s). A maximum cut-off time of 90 s was applied, and mice sustained on the hanging wire beyond the cut-off time were given a fall latency score of 90 s.

### Pole test

The pole test was included in this study to evaluate bradykinesia and motor coordination impairments commonly associated with dopaminergic dysfunction. Mice were softly held and placed with their heads facing upward at the top of a wooden pole measuring 8 mm in diameter and 50 cm long. The wooden bar was positioned vertically and attached to a base with sawdust in order to avoid injury in case of a potential fall. Animals’ time taken to rotate itself downward completely is denoted as T-Turn, and the time taken to run down to the base of the pole is denoted as the T-total. A maximum cut-off time of 120 s was allocated to the animals. Animals that failed to perform pole test tasks within the maximum cut-off time were assigned the maximum cut-off time of 120 s. Mice were subjected to 4 days of training on how to descend the pole. Elongation of either T-Turn or T-total is a measure of bradykinesia manifestation.

### Biochemical assays

#### Tissue preparation

On Day 45 of the experiment, approximately 3 h post-final oral gavage, following the conclusion of the neurobehavioral assessments, brain tissues were collected. Mice were anesthetized with intraperitoneal (i.p.) injection of ketamine (90 mg/kg) and xylazine (7 mg/kg) before they were euthanized. Doses of ketamine and xylazine used in this study were chosen based on the experience in our laboratory and fall within the reported doses for mice (Naples et al., 2020; Hidayat and Wulandari, 2021). After anesthesia, mice were euthanized, and their brains were quickly collected. Midbrain areas chosen for biochemical analysis were immediately dissected and isolated based on anatomical landmarks in accordance with the Mouse Brain Atlas (Paxinos and Franklin, 4th edition). The brain tissue harvested from the area corresponds to bregma −2.80 mm to −3.80 mm, comprising the substantia nigra and ventral tegmental area. In order to ensure standardization, precisely 0.5 g of brain tissue dissected from the midbrain area was weighed for each animal. In the events where the midbrain weighed less than 0.5 g, adjacent uniform sections contained in the coordinate range were added to standardize the weight. The 0.5 g tissue was quickly homogenized in 3 mL of 0.1 M ice-cold phosphate buffer, pH 7.4, using a Percellys Minilys Homogenizer (Cayman Chemical, United States) for 60 s. The homogenate was thereafter centrifuged at 5000 rmp for 15 min, and the supernatant was separated, aliquoted into sterile Eppendorf tubes, and stored at −80 °C until further multiple biochemical analyses, including ELISA.

#### Measurement of lipid peroxidation in the midbrain

As previously described by Ohkawa et al. (1979), the concentrations of thiobarbituric acid reactive substance (TBARS) were used as an indication of malondialdehyde (MDA) production. MDA is a final product of lipid peroxidation and complexes with TBA-TCA, forming a colored reaction at high temperature, showing as an absorption maximum at 535 nm. Normal saline of 100 μL was reacted with 100 μL of supernatant along with 400 μL of TBA–TCA mixture, followed by incubation for 10 min in a boiling water bath and then allowed to cool at room temperature. After centrifugation at 500 rpm for 10 min, 100 μL of supernatant was reacted with 100 μL of 0.7% TBA in a cuvette and read at 535 nm. The concentration of MDA was calculated using a standard curve from 1, 1, 3, 3-tetraethoxypropane and expressed in nmol/mg.

#### Measurement of catalase activity

Catalase activity was estimated by mixing 0.05 mL tissue supernatant with 3 mL of hydrogen peroxide (H_2_O_2_). The absorbance activity was calculated against a blank that contained 3 mL of PBS at a wavelength of 240 nm. The quantity of H_2_O_2_ was relative to the absorbance, which was reduced when catalase inhibited H_2_O_2_. This is an estimation of H_2_O_2_ breakdown and is given as mol H_2_O_2_ broken down per milligram of protein per minute (Condon et al., 1982).

#### Measurement of superoxide dismutase activity (SOD)

Superoxide dismutase activity (SOD) was evaluated using the nitroblue tetrazolium (NBT) as previously described by (Sun et al., 1988). Concisely, 50 μL of the crude enzyme extract was mixed with a solution containing 13 mM L -methionine, 75 μM p-nitro blue tetrazolium chloride (NBT), 100 μM EDTA, and 2 μM riboflavin in a 50 mM potassium phosphate buffer (pH 7.8). The reaction took place in assay tubes under 30 W fluorescent lamp illumination at 25 °C for 15 min. Using a spectrophotometer, the blue formazane produced by NBT photo-reduction was calculated through the absorbance at 620 nm. There was no enzyme extract in the control reaction mixture. The blank solution was stored in the dark and composed of the same complete reaction solution. One SOD unit of activity was denoted as the quantity of enzyme required to inhibit 50% of NBT photo-reduction relative to tubes lacking the tissue extract. Activity was measured as units (U) per mg soluble protein per min (U mg^−1^ protein min^−1^).

#### Measurement of glutathione peroxidase (GPx) activity

Glutathione oxidation by the GPx cumene hydroperoxide is catalyzed by the GPx. The oxidized glutathione is converted back to regenerative glutathione in the presence of glutathione reductase and NADPH. In this study, the NADP^+^ obtained at 340 nm was assessed.

#### Enzyme-linked immunosorbent assay (ELISA)

The ELISA assays were conducted for NF-κB, dopamine, and proinflammatory cytokines, including TNF-α, IL-1β, and IL-6, in accordance with the manufacturer’s instructions using mouse-specific ELISA kits from Elabscience. Total protein concentrations were determined using the Bradford/bicinchoninic acid (BCA) assay following homogenization of the standardized 0.5 g midbrain tissue, centrifugation at 5,000 rmp for 15 min, and division of the supernatants into aliquots. A specific quantity of supernatant –ranging from 50 μL to 100 μL, conditioned to Elabscience’s kit protocol was added to each well for each ELISA assay. For instance, 50 μL of supernatant was used for the dopamine assay, while 100 μL was used for each of the NF-κB, TNF-α, IL-1β, and IL-6 assays. These quantities of samples corresponded to known concentrations of proteins as established by the BCA assay. In order to ensure consistency across endpoints, the same animals and the same midbrain homogenate samples were used for the biochemical analysis. Protein samples were incubated with the corresponding antibodies in 96-well plates, and absorbance values were read using a microplate reader. Final concentrations for each analyte were normalized to protein concentration.

#### Immunohistochemistry

After the animals for histology study were euthanized, 0.9% normal saline was used to perfuse the mice transcardially, subsequently by 4% paraformaldehyde in order to fix the tissues. The brains were gently and carefully removed from the skull, and the substantia nigra was dissected out and stored in 4% paraformaldehyde for 24 h, thereafter the tissues were transferred to 0.1 M phosphate-buffered saline (PBS) overnight for rinsing and removal of excess paraformaldehyde, after which they were dehydrated through subjection to a graded series of ethanol, cleared xylene, and paraffin-embedded. The tissue slices of about 4–5 μm thick were fixed on saline-coated slides, and deparaffinized twice with the aid of xylene. Thereafter, they were rehydrated in 100, 75, and 50% anhydrous alcohol. Antigen retrieval was performed through heating in sodium citrate buffer, Tween 20 at pH 6.0 for 20–40 min. The sections were incubated using 3% H2O2 for 20–30 min at room temperature to reduce endogenous peroxidase. After washing with PBS, the sections were incubated with anti-tyrosine hydroxylase (Sigma T8700) at 1:1000 overnight at 4 °C. After the sections were rinsed with PBS, they were incubated with polyclonal goat anti-rabbit immunoglobulins/HRP (Dako P0448) 1:100 for 1 h and washed in PBS with Triton X-100 for 10 min. Thereafter, the sections were visualized with diaminobenzidine (DAB). The color changed to brown within 3–5 min. The sections were then dyed with hematoxylin, washed with deionized water, dried, and sealed with sealing solution. Finally, the slides were appropriately coded before a light microscope (Leica DM 500, Germany) examination and image capturing with the aid of a digital camera (Leica ICC50 E, Germany). TH + neuronal counts were not quantified because of the unavailability of an unbiased stereological technique and the use of thin sections. Observations were, however, made across groups on qualitative comparison of TH immunoreactivity so as to allow comparative interpretation of TH expression patterns.

### Statistical analyses

Behavioral test data as well as biochemical results were compared between the treated and control groups. Statistical analysis of data was performed through one-way analysis of variance (ANOVA). Normality of the data distribution was evaluated in order to ensure the validity of the parametric test. In the case of different independent variables, a statistical analysis of the data was conducted using two-way ANOVA, followed by a Tukey *post hoc* test for multiple comparisons. The results were presented as mean ± S. E. M with the *p* < 0.05 value being considered statistically significant. All statistical analyses were conducted using GraphPad PRISM 9 software (GraphPad PRISM 9.1.1 Software, San Diego, CA, United States).

## Results

### Open field maze test

**Figure 2:**
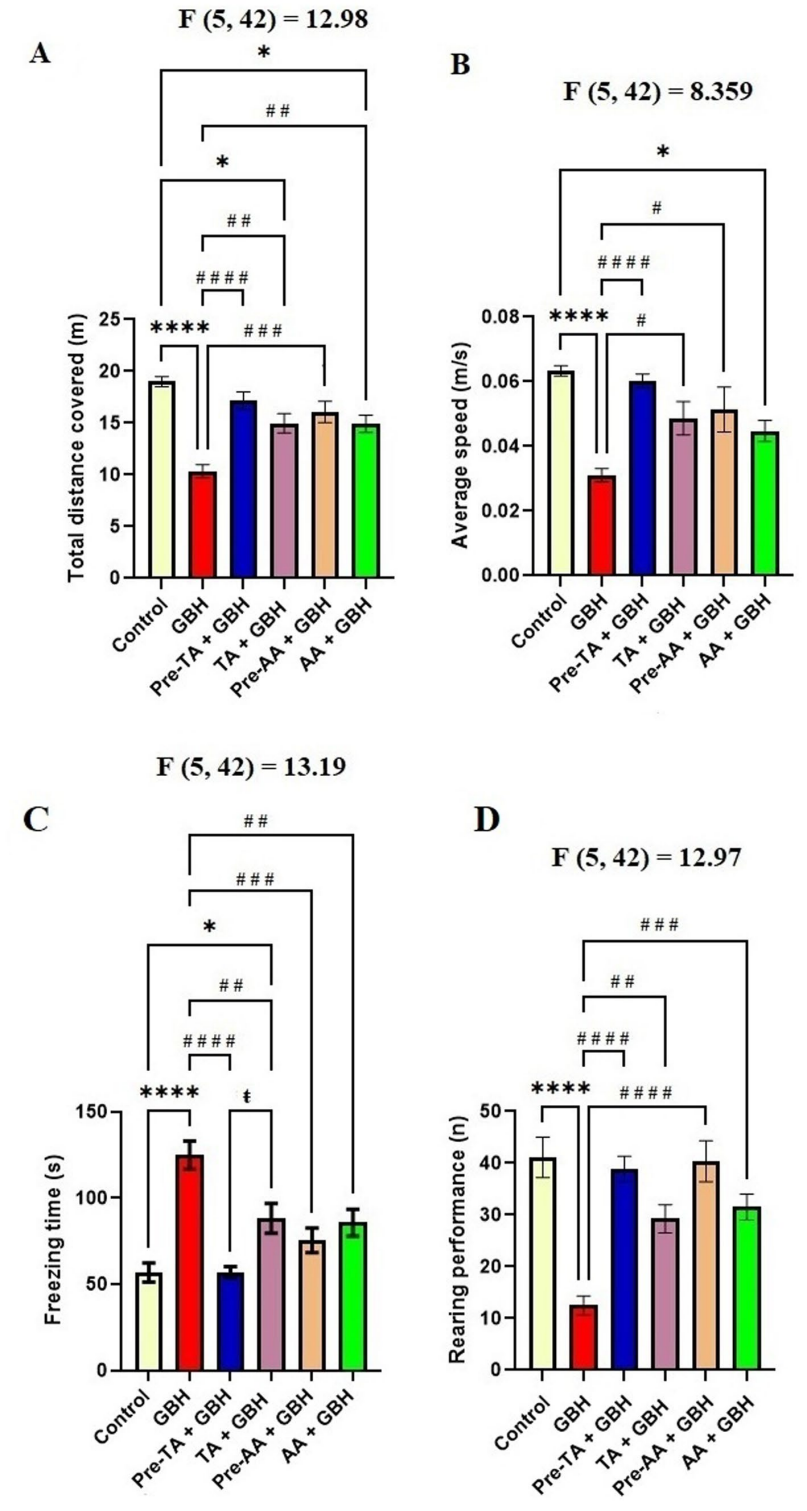
TA protected against motor dysfunction in mice exposed to GBH. **(A)** Total distance covered in the open field test. **(B)** Average speed of movement in the open field test. **(C)** Freezing time in the open field maze. **(D)** Rearing performance in the open field maze. Data are reported as mean ± S. E. M (*n* = 8 animals per group). *p < 0.05, **p < 0.01, ***p < 0.001, ****p < 0.0001 versus Control; ^#^*p* < 0.05, ^##^*p* < 0.01, ^###^*p* < 0.001, ^####^*p* < 0.0001 versus GBH; ^ŧ^*p* < 0.05, ^ŧŧ^*p* < 0.01, ^ŧŧŧ^*P* < 0.00, ^ŧŧŧŧ^*P* < 0.0001versus Pre-TA + GBH. Statistical analysis was performed using one-way ANOVA followed by Tukey’s *post hoc* multiple comparison test.

Figures 2A–D shows the results of the effects of TA pre-treatment or TA co-administered with GBH on motor functions in mice exposed to GBH. As observed in the open field maze test, mice exposed to GBH alone manifested a significantly (*p* < 0.0001) decreased total distance covered as well as decreased average speed of movement when compared to the control group, suggesting decreased motor function (Figures 2A,B). On the contrary, the Pre-TA + GBH group displayed a significant increase (*p* < 0.0001) in the total distance covered and average speed of movement compared to the GBH group. This effect was similar to that of the control and AA-treated groups. Furthermore, the TA + GBH group also showed a significantly increased total distance covered (*p* = 0.028) and average speed of movement (*p* = 0.0368) as compared to the GBH group. According to Figures 2C,D, the duration with which mice were completely stationary was defined as the ‘freezing time,’ and the frequency with which the animals stood on their hind limbs and against the wall of the open field maze was defined as the ‘rearing performance’. An increased freezing duration, essentially with ‘stretch attempts’ and decreased rearing performance, is a manifestation of an increased level of anxiety. According to the open field test, it was observed that the group exposed to GBH alone, showed a significantly (*p* < 0.0001), increased freezing time relative to the control (*p* < 0.0001), Pre-TA + GBH (*p* < 0.0001), TA + GBH (*p* = 0.0070), Pre-AA + GBH (*p* = 0.0001), and AA + GBH (*p* = 0.0034) groups. Similarly, the rearing activity of the GBH group was observed to be significantly low when compared to the control (*p* < 0.0001), Pre-TA + GBH (*p* < 0.0001), TA + GBH (*p* = 0.0039), Pre-AA + GBH (*p* < 0.0001), and AA + GBH (*p* = 0.0008) groups.

**Figure 3:**
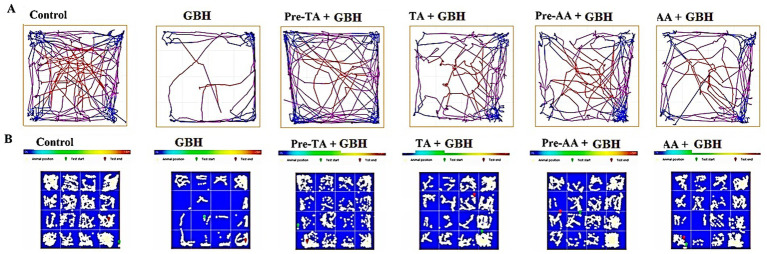
Influence of TA on exploratory profiles in GBH-exposed mice during the 5 min trial in an open field maze apparatus. **(A)** Track plots; **(B)** Occupancy plots. The data were analyzed using video-tracking software (*n* = 8 animals per group) (ANY-maze, Stoelting CO, USA).

Furthermore, mice exposed to GBH alone manifested exploratory insufficiencies as verified by a marked reduction in the track plots density (Figure 3A) and occupancy plots (Figure 3B) with prolonged time utilized in a particular region of the open field maze. Conversely, the group pre-treated with TA (Pre-TA + GBH) and the TA and GBH co-treated group (TA + GBH) evidently abated GBH-mediated exploratory insufficiencies by increasing the track plot density and decreasing the occupancy density in the occupancy plots in comparison with the GBH-exposed group. These effects were also observed to be similar to the results obtained in the control and mice treated with AA.

### Narrow beam walk test

**Figure 4:**
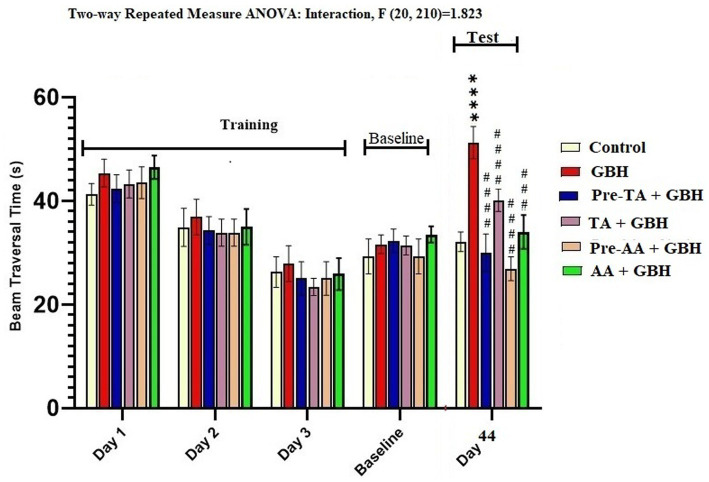
TA increased beam traversal time during the beam walk test of GBH-exposed mice. Data are reported as mean ± S. E. M (*n* = 8 animals per group). ^*^p < 0.05, **p < 0.01, ***p < 0.001, ****p < 0.0001 versus control; ^#^*p* < 0.05, ^##^*p* < 0.01, ^###^*p* < 0.001, ^####^*p* < 0.0001 versus GBH. Statistical analysis was performed using one-way ANOVA followed by Tukey’s post hoc multiple comparison test.

Results from the narrow beam walk test, according to Figure 4, revealed that GBH-exposed mice manifested bradykinesia as the time to traverse the beam in this group was significantly (*p* < 0.0001) reduced when compared to the animals in the control, Pre-TA + GBH, and AA-treated groups. On the contrary, the traversal time of the mice in the TA + GBH group was not significantly different from that of the GBH group. Importantly, the beam traversal times across the groups during the training days and the baseline performance test were not significantly different.

### Pole test

**Figure 5:**
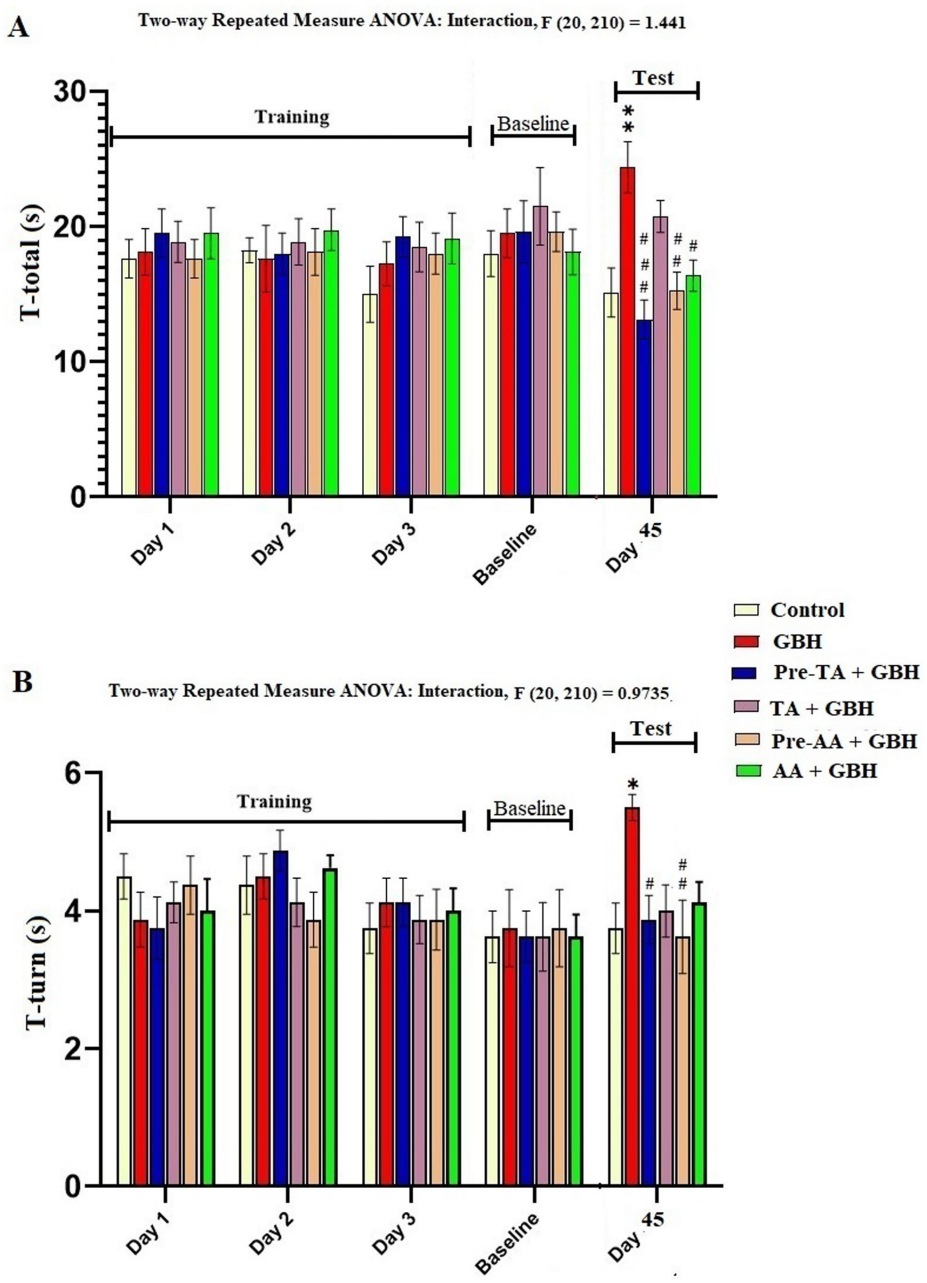
Effect of TA treatment on motor function in the pole test. **(A)** GBH-induced bradykinesia and its inhibition by TA in the pole test. **(B)** GBH-induced prolongation of rotation time was inhibited by TA in the pole test. Data are reported as mean ± S. E. M (*n* = 8 animals per group). *p < 0.05, **p < 0.01, ***p < 0.001, ****p < 0.0001 versus Control; ^#^*p* < 0.05, ^##^*p* < 0.01, ^###^*p* < 0.001, ^####^*p* < 0.0001 versus GBH. Statistical analysis was performed using one-way ANOVA followed by Tukey’s post hoc multiple comparison test.

Animals were suspended in a headward position at the top of the pole and allowed to rotate downward and descend to the base of the cage. The animal’s time taken to rotate itself downward completely is denoted as T-Turn (Figure 5A), and the time taken to run down to the base of the pole is denoted as the T-total (Figure 5B). In this test, the control mice displayed a significantly (*p* = 0.0029) reduced T-total and T-turn (*p* = 0.0199) when compared to the GBH group. It was also observed that the GBH group manifested significantly increased T-total (*p* < 0.0001) and T-turn (*p* = 0.0389) values relative to the Pre-TA + GBH group. Essentially, the inhibition of GBH-induced prolonged T-total and T-turn through pre-treatment with TA was not statistically different relative to the control and pre-treatment with AA. This is suggestive of the anti-bradykinesia effect of TA pre-treatment in GBH exposure in mice. Worthy of note is that there was no statistical difference in the T-total and T-turn values across the groups during the training days and baseline performance test.

### Hanging wire test

**Figure 6:**
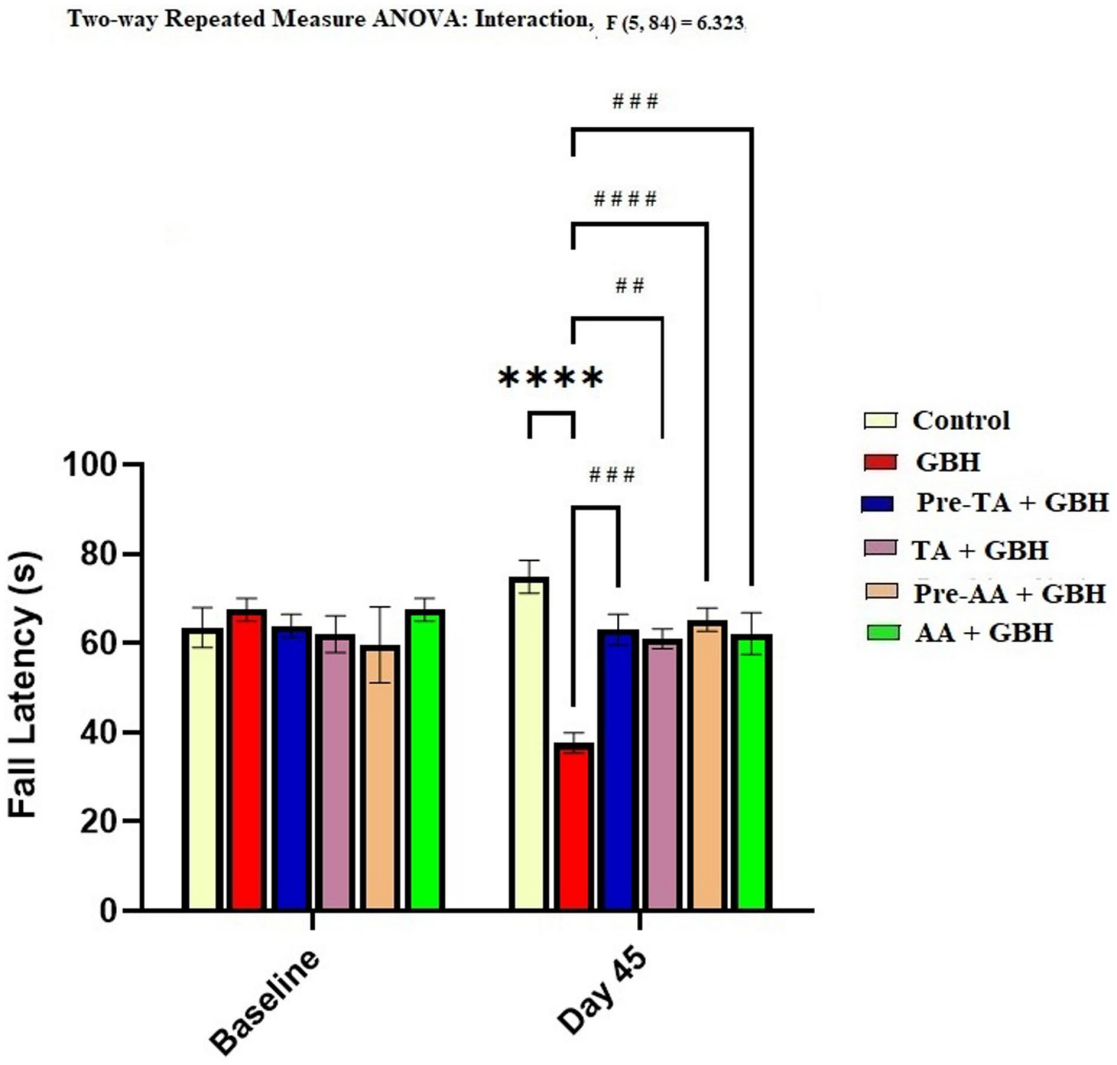
GBH-induced reduction in fall latency time and inhibited by TA in the hanging wire test. Data are reported as mean ± SEM (*n* = 8 animals per group). ^*^p < 0.05, ^**^p < 0.01, ^***^p < 0.001, ^****^p < 0.0001 versus control; ^#^*p* < 0.05, ^##^*p* < 0.01, ^###^*p* < 0.001, ^####^*p* < 0.0001 versus GBH. Statistical analysis was performed using one-way ANOVA followed by Tukey’s post hoc multiple comparison test.

The forelimb hanging wire test was used in this study to assess the neuromuscular strength of mice exposed to GBH. Results (Figure 6) showed that the GBH group displayed a significant (*p* < 0.0001) reduction in the fall latency compared to the control on the 45th day of the experiment. Conversely, the Pre-TA + GBH group manifested a significant (*p* = 0.0003) increase in the fall latency on the 45th day of the experiment as compared to the GBH group. This effect was not significantly different from the control and the AA-treated groups. In addition, the TA + GBH group also exhibited a significant (*p* = 0.002) increase in the hanging time relative to the GBH group. Notwithstanding, there was no significant alteration in the fall latency of the mice across the groups during the baseline performance. Put together, these results showed that the GBH exposure protocol used in this study induces motor deficits, a behavioral phenotype typical of PD animal models.

### Malondialdehyde evaluation

**Figure 7:**
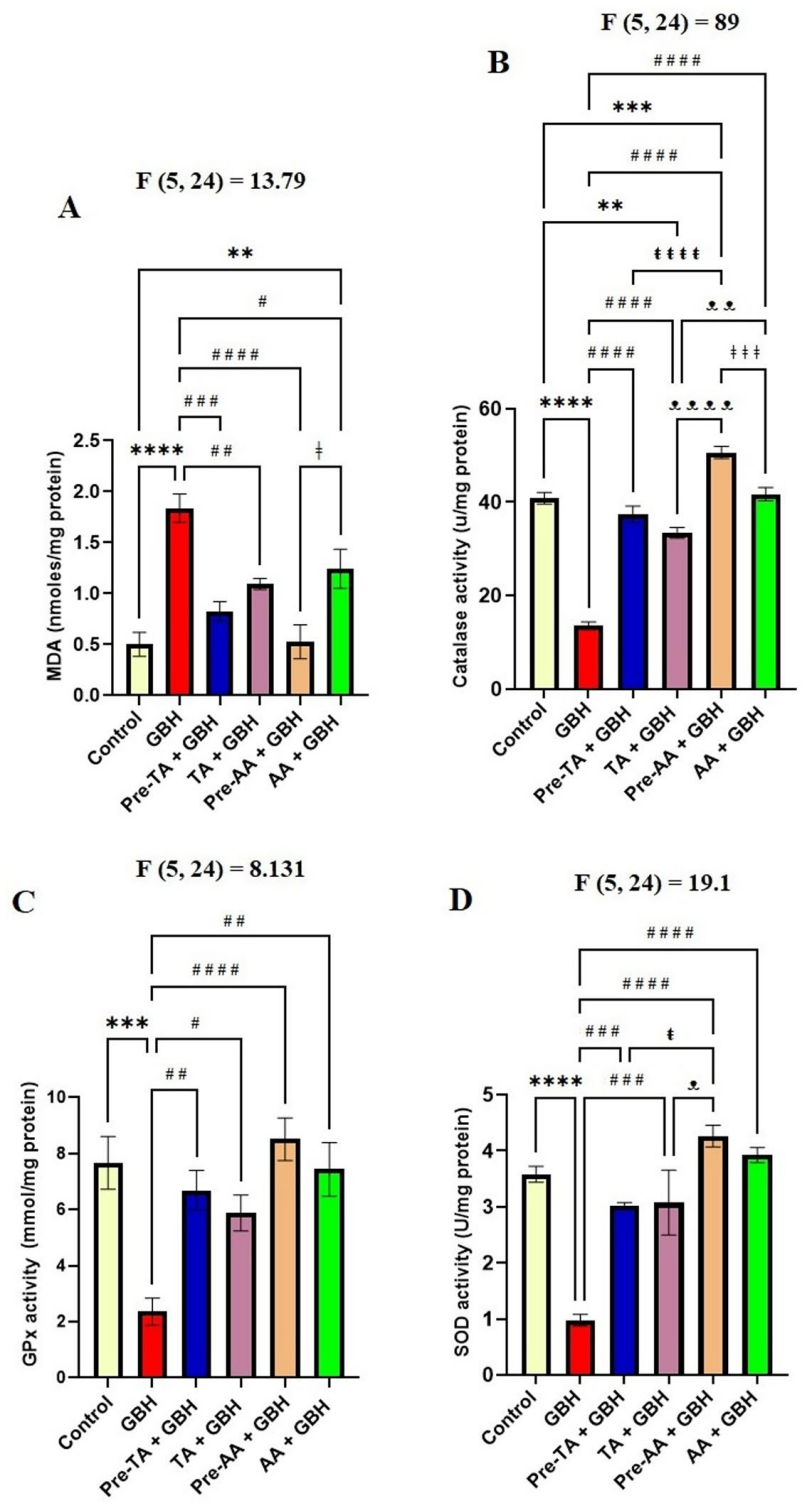
TA prevented lipid peroxidation and increased activities of antioxidant enzymes in the brain of mice exposed to GBH. **(A)** Midbrain MDA levels. **(B)** Midbrain catalase antioxidant enzyme activity. **(C)** Midbrain GPx antioxidant enzyme activity. **(D)** Midbrain SOD antioxidant enzyme activity. Data are reported as mean ± S. E. M (*n* = 5 animals per group). *p < 0.05, **p < 0.01, ***p < 0.001, ****p < 0.0001 versus Control; ^#^*p* < 0.05, ^##^*p* < 0.01, ^###^*p* < 0.001, ^####^*p* < 0.0001 versus GBH; ^ŧ^*p* < 0.05, ^ŧŧ^*p* < 0.01, ^ŧŧŧ^*P* < 0.00, ^ŧŧŧŧ^*P* < 0.0001 versus re-TA + GBH; ^ᴥ^*p* < 0.05, ^ᴥᴥ^*p* < 0.01, ^ᴥᴥᴥ^*P* < 0.001, ^ᴥᴥᴥᴥ^*P* < 0.0001 versus cTA + GBH. ^ǂ^p < 0.05, ^ǂǂ^*p* < 0.01, ^ǂǂǂ^*p* < 0.001, ^ǂǂǂǂ^*p* < 0.0001 versus Pre-AA + GBH. Statistical analysis was performed using one-way ANOVA followed by Tukey’s *post hoc* multiple comparison test.

Malondialdehyde (MDA) is a marker of lipid peroxidation. The levels of MDA were measured in the midbrain as demonstrated in Figure 7A. It was observed that GBH exposure caused an increase in lipid peroxidation levels of MDA in the midbrain, which was significantly (*p* < 0.0001) increased in the GBH group relative to the control. However, the Pre-TA + GBH group manifested a significant (*p* = 0.0003) reduction in the concentrations of MDA in the midbrain, respectively, with respect to the GBH group. Worthy of note is that this effect was not significantly different when compared to the control and the AA-treated groups. Moreover, co-administration of TA with GBH depicts ameliorative effects with a significant (*p* = 0.0085) reduction in the concentrations of MDA in the midbrain relative to the GBH group. Based on these data, it can be inferred that both pre-administration of TA and co-administration of TA with GBH during GBH exposure have both neuroprotective and ameliorative effects against lipid peroxidation in the midbrain.

### Evaluation of catalase antioxidant enzyme activity

The effect of GBH exposure on catalase antioxidant enzyme activities in the midbrain was also assessed. It was noted that the GBH group displayed a significant (*p* < 0.0001) reduction in catalase activity in the midbrain in comparison with the control group. On the contrary, the Pre-TA + GBH group presented with a significant (*p* < 0.0001) increase in the activity of catalase enzyme in the midbrain with respect to the GBH group. Of note is that this action was not significantly different in comparison with the control group (Figure 7B). The effects of the TA + GBH group were observed to follow the pattern of the Pre-TA + GBH group.

### Evaluation of glutathione peroxidase antioxidant enzyme activity

Data according to Figure 7C revealed that in the midbrain, the activity of GPx enzyme was significantly (*p* = 0.0007) reduced in the GBH group when compared with the control group. Similarly, Pre-TA + GBH revealed a remarkable increase in the activity of GPx enzyme in the midbrain as compared to the GBH group. An effect observed to be similar to the control and the AA-treated groups. The TA + GBH group also exhibited a significant (*p* = 0.0063) increase in the activity of GPx enzyme in the midbrain in comparison with the GBH group. Data obtained from this study is a reflection of oxidative stress taking place in the brain regions of GBH-exposed mice. Nevertheless, pre-treatment of animals with TA before and co-administration of TA with GBH were able to proffer neuroprotective as well as ameliorative functions.

### Evaluation of superoxide dismutase antioxidant enzyme activities

Superoxide Dismutase (SOD) enzyme is one of the powerful antioxidants whose activities were observed to be significantly (*p* < 0.0001) reduced in the midbrain of the GBH group when compared to the control group. Pre-TA + GBH, however, displayed a significant increase (*p* = 0.0002) in activity of SOD enzyme in the midbrain with respect to the GBH group. The values obtained in this group were not significantly different relative to the control and the AA-treated groups. Furthermore, the results obtained from the TA + GBH group revealed a significant (*p* = 0.0001) increase in the activity of SOD enzyme in the midbrain in comparison with the GBH group (Figure 7D).

### Evaluation of nuclear factor kappa B (NF-κB) levels

**Figure 8:**
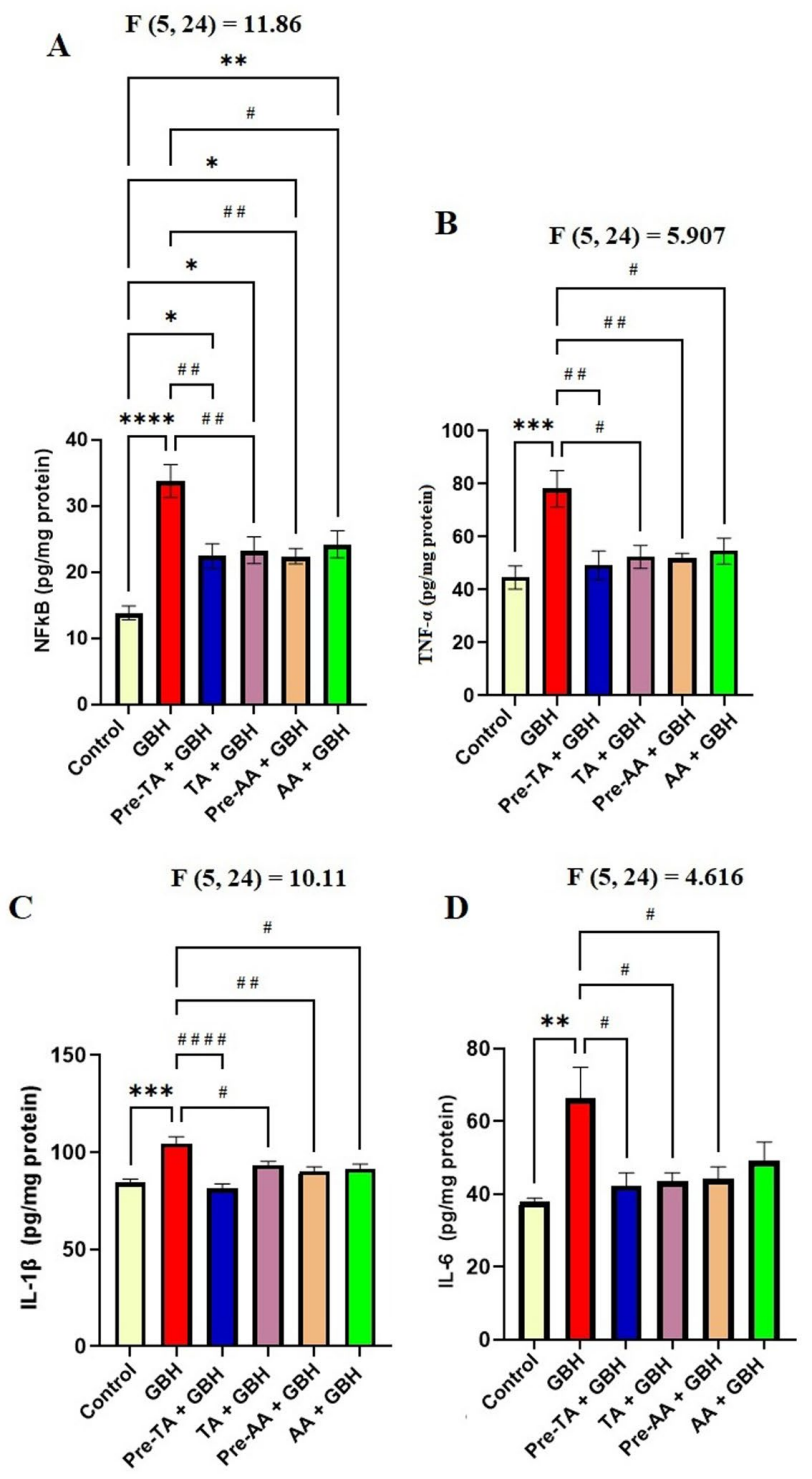
Effects of TA on inflammatory mediators in the midbrain of in the brain of mice exposed to GBH. **(A)** Midbrain NF-κB concentration **(B)** Midbrain TNF-α concentration. **(C)** Midbrain IL-1β concentration. **(D)** Midbrain IL-6 concentration. Data are reported as mean ± S. E. M (*n* = 5 animals per group). *P < 0.05, **P < 0.01, ***p < 0.001, ****p < 0.0001 versus Control; ^#^*P* < 0.05, ^##^*P* < 0.01, ^###^*p* < 0.001, ^####^*p* < 0.0001 versus GBH. Statistical analysis was performed using one-way ANOVA followed by Tukey’s *post hoc* multiple comparison test.

Data on the influence of TA pre-treatment and co-administration with GBH on the inflammatory markers during GBH exposure are presented in Figure 8A. In comparison with the control group, the GBH group exhibited a significantly (*p* < 0.0001) increased concentration of NF-κB in the midbrain. In contrast to this observation, the Pre-TA + GBH group manifested a considerable (*p* = 0.0027) reduction in the levels of NF-κB in the midbrain compared to the GBH group. These values were significantly (*p* < 0.0316) increased in comparison with the control, but not significantly different when compared with the AA groups. Similarly, TA + GBH displayed a significant (*p* = 0.0060) reduction in the concentrations of NF-κB in the midbrain. This effect was significantly (*p* = 0.0148) reduced with respect to the control group but not significantly different relative to the AA-treated groups.

### Evaluation of tumor necrosis factor-alpha (TNF-α) levels

The effects of TA on tumor necrosis factor-alpha (TNF-α) levels were evaluated in the midbrain. It was observed that the GBH group displayed a significant increase in the levels of TNF-α in the midbrain (*p* = 0.0007) when compared with the control group. Remarkably, TA pre-treatment exhibited a significant reduction in the concentration of TNF-α in the midbrain (*p* = 0.0036) when compared with the GBH group. Indeed, these values were not significantly different from the control and AA-treated groups. The TA + GBH group also displayed a significant (*p* = 0.0112) reduction in the concentrations of TNF-α in the midbrain in comparison with the GBH group. These effects were also not significantly different when compared with the AA-treated groups, but significant alterations were observed when compared with the control (Figure 8B).

### Evaluation of interleukin-1β (IL-1β) concentrations

It was also verified according to Figure 8C that chronic exposure to GBH caused an increased concentration of Interleukin-1β (IL-1β) in the midbrain. Specifically, the GBH group displayed a significant (*p* < 0.0001) increase in the concentrations of IL-1β in the midbrain relative to the control group. This effect was inhibited in the group pre-treated with TA, with a significant (*p* < 0.0001) reduction in the concentrations of IL-1β in the midbrain in comparison with the GBH group. Worthy of note is that these values were not significantly different when compared with the control and the AA-treated groups. The TA + GBH group also manifested a remarkable (*p* = 0.0454) reduction in the concentrations of IL-1β in the midbrain relative to the GBH group. Based on the gathered results, it can be inferred that TA has an anti-IL-1β effect during a chronic GBH exposure in mice.

### Evaluation of interleukin-6 (IL-6) concentrations

Results on the effects of chronic GBH exposure showed a significant (*p* = 0029) increase in the concentrations of Interleukin-6 (IL-6) in the midbrain of the GBH group with respect to the control group. Pre-TA + GBH demonstrated a significant reduction in the concentrations of IL-6 in the midbrain (*p* = 0.0152) compared with the GBH group. This effect was not significantly different when compared with the control and the AA-treated groups. In addition, the TA + GBH group revealed a significant (*p* = 0.0221) reduction in the concentrations of IL-6 in the midbrain relative to the GBH group (Figure 8D).

### Effect of tannic acid on dopamine levels in the brain of mice exposed to GBH

**Figure 9:**
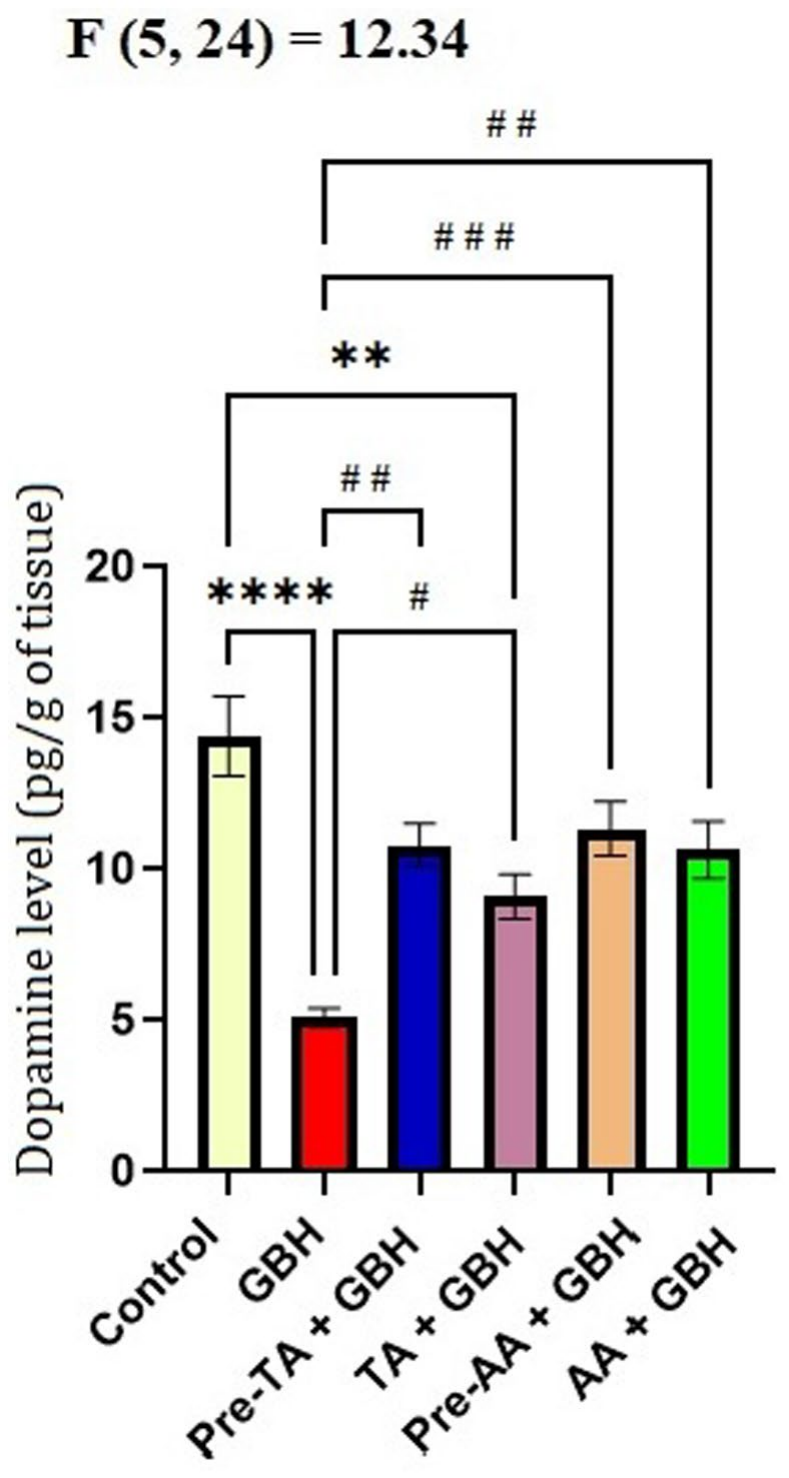
TA increased the concentrations of dopamine in the brains of mice exposed to GBH. Data are reported as mean ± S. E. M (*n* = 5 animals per group). *P < 0.05, **P < 0.01, ***p < 0.001, ****p < 0.0001 versus control; ^#^*P* < 0.05, ^##^*P* < 0.01, ^###^*P* < 0.001, ^####^*P* < 0.0001 versus cGBH. Statistical analysis was performed using one-way ANOVA followed by Tukey’s post hoc multiple comparison test.

Data from Figure 9 revealed that there was a significant (*p* < 0.0001) reduction in the concentration of dopamine in the midbrain of the GBH group in comparison with the control group. Conversely, the Pre-TA + GBH group displayed an inhibition of a decrease effect with a significant increase in the concentrations of dopamine in their midbrain (*p* = 0013). Notably, these values were not significantly different compared to the control and the AA groups. Results from the TA + GBH group also showed a significant (*p* = 0.0358) increase in the levels of dopamine in their midbrain relative to the GBH group. Notably, the effects of co-administration of TA and GBH (TA + GBH group) were similar to those of the control and the AA groups in the midbrain.

### Tannic acid prevented dopaminergic neuronal loss

**Figure 10:**
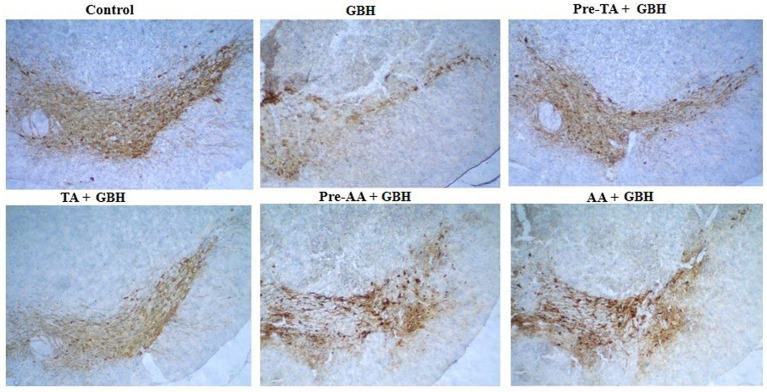
Representative photomicrographs of mouse brain cross sections showing the tyrosine hydroxylase (TH)-immunoreactive neurons at the substantia nigra pars compacta (SNpc) of TA-treated groups following GBH exposure. Qualitative assessment of the sections was performed (scale bar = 100 μm).

Synthesis of dopamine requires the activity of the rate-determining enzyme, tyrosine hydroxylase (TH), in the substantia nigra pars compacta. A visible reduction in TH immunoreactivity was noticed in the substantia nigra pars compacta of mice of the GBH group relative to the control. TA, treatment appeared to increase TH staining intensity in a similar pattern to AA treatments (Figure 10).

## Discussion

Emerging evidence demonstrating the potential neurotoxic effects of the environmental factors, including chronic GBH exposure, has stimulated progressive research attention (Costas-Ferreira et al., 2022). Reports from animal studies have shown that chronic exposure to glyphosate disrupts neuronal homeostasis through the generation of reactive oxygen species, mitochondrial dysfunction, disrupted energy biogenesis, and neuroinflammatory activation (Qi et al., 2023; Iqubal et al., 2020), processes that contribute to dopaminergic neurotransmission impairment and motor dysfunction. In agreement with these reports, the present study demonstrated that chronic exposure to GBH in rats induced motor dysfunction associated with midbrain oxidative stress, increased concentrations of pro-inflammatory cytokines, reduced concentrations of dopamine, and altered tyrosine hydroxylase (TH) immunoreactivity within the SNpc. These alterations were attenuated with administration of TA, suggesting neuroprotection against GBH-induced motor dysfunction and the associated dopaminergic neurotoxicity.

Neurobehavioral evaluations from open field, pole, narrow beam walk, and hanging wire tests showed considerable impairments in motor functions as reflected in poor locomotor activity, coordination, and muscular endurance after chronic GBH exposure in rats. These findings agree with the previous reports (Ait Bali et al., 2017; Hernández-Plata et al., 2015; Ait-Bali et al., 2020), revealing reduced locomotion, hypoactivity, and elevated levels of anxiety and depression-like behavior following sub-chronic or chronic glyphosate exposure. Similar reports involving dichlorvos herbicide (Sun et al., 2006), methyl parathion organophosphate (Bushnell et al., 1990), and diisopropylfluorophosphate insecticide have demonstrated comparable motor impairments as observed in the present study, supporting the assertion that environmental neurotoxicant exposure to environmental toxicants usually induces motor dysfunction by interfering with the dopaminergic signaling (Cohen, 2000). Notably, administration of TA improved significantly the behavioral performance across the paradigm, suggesting functional motor output preservation despite exposure to the GBH. This observation corroborates previous findings indicating behavioral improvement after administration of antioxidant polyphenol in toxin-induced neurotoxicity models (Singh et al., 2017).

Oxidative stress emerged as a pivotal mechanism underpinning GBH-induced neurotoxicity (De Batista et al., 2023). An increase in the concentrations of the midbrain malondialdehyde (MDA) alongside reductions in the activities of endogenous antioxidant enzymes, including SOD, GPx, and catalase, in rats exposed to GBH indicates dyshomeostasis within the midbrain. Gallegos et al. (2018) reported a comparable oxidative alteration in experimental paradigms where glyphosate formulations elevated the generation of reactive oxygen species and lipid peroxidation in neural cells. TA administration restoring the activities of antioxidant enzymes reinforces the previous report showing potent free radical scavenging properties of TA and other polyphenolic compounds. Other reports, including rotenone- and lipopolysaccharide-induced neurotoxicity, have shown similar antioxidant restoration of TA (Azimullah et al., 2023; Basu et al., 2018; Ullah et al., 2022), suggesting that reinforcement of endogenous antioxidant defenses represents a concerted protective mechanism of TA.

There is a significant contribution of neuroinflammatory signaling to the observed neurotoxicity in the present study. Chronic GBH exposure induced an increased concentration of TNF-α, IL-1β, IL-6, and NF-κB activation within the midbrain, indicating activation of the inflammatory pathway. This is consistent with previous studies demonstrating that glyphosate formulation promotes the release of cytokines and microglia activation (Winstone et al., 2022; Izumi et al., 2024; de Castro Vieira Carneiro et al., 2023). Administration of TA considerably reduced the concentrations of these inflammatory mediators, corroborating a previous report from experimental neuroinflammation models (Wu et al., 2019), where TA suppresses NF-κB–dependent inflammatory pathways. Similar anti-inflammatory action of TA has been revealed in traumatic brain injury and metabolic neurodegenerative paradigms (Salman et al., 2020; Gerzson et al., 2020), supporting the concept that TA-mediated neuroprotection is orchestrated through modulation of inflammatory signaling.

The homeostasis of dopamine was further evaluated through quantification of dopamine concentrations and TH immunohistochemistry. Exposure to GBH reduced the concentrations of dopamine and decreased TH immunoreactivity within the SNpc, suggesting the disruption of the dopaminergic neuronal system. Comparatively, a previous report has demonstrated reductions in dopaminergic markers following chronic exposure to pesticides (Ait-Bali et al., 2020), strengthening the idea of nigrostriatal pathway vulnerability to environmental neurotoxicants. The capacity of TA treatment to preserve TH-positive neurons after GBH exposure is in agreement with previous reports demonstrating that polyphenolic antioxidants attenuate toxin-induced dopaminergic neurotoxicity. The present study dissuades from giving a definitive prevention of dopaminergic neuronal loss because stereological quantification was not performed; nonetheless, the aggregation of neurochemical and behavioral improvements contributes to the functional neuroprotection observed in this study.

Mechanistically, the present findings suggest that TA primarily mitigates upstream toxic processes triggered by GBH exposure. The concurrent reduction of oxidative stress and inflammatory signaling indicates interruption of mutually reinforcing pathological pathways known to compromise neuronal function. Similar upstream protective actions have been proposed in studies demonstrating that TA attenuates rotenone-induced dopaminergic toxicity through suppression of oxidative and inflammatory cascades. However, the current data do not distinguish whether TA prevents initial cellular injury or promotes recovery following toxic insult, and further studies incorporating temporal analyses will be required to clarify these mechanisms.

An additional consideration is that pure glyphosate was not used in this study; rather, the present study utilized the commercial GBH formulation, containing surfactants and adjuvants capable of enhancing penetration, stability, and toxicity relative to pure glyphosate. This paradigm is relevant as the commercial formulations are exposed to rather than the pure glyphosate. Therefore, the motor deficits and neurochemical alterations observed in this study may not be solely attributed to glyphosate itself, even though these additives are considered inert and non-toxic (Zaller et al., 2021; Mesnage et al., 2022). Instead, the findings noticed in this study reflect the aggregated biological activity of the complete commercial formulation, which is ideal as it represents real-world exposure. The dose of glyphosate used in this study was (500 mg/kg/day), a dose higher than the usual environmental exposures. Needful to state that this dose was used as such, which is commonly used in toxicological research to identify the possible neurotoxic effects and overcome challenges associated with differences in metabolism. For instance, rodents have the capacity to metabolize xenobiotics more quickly than humans, and high-dose exposure assists in modeling cumulative or chronic real-life exposure situations, most importantly for people who are at risk of occupational contact, including farmers and pesticide users. Hence, the dose applied in this study gives a ‘worst-case’ or hazard recognition strategy rather than a direct simulation of average human environmental exposure (Borgert et al., 2021). The major limitation of this study is the use of only male mice. We acknowledge possible sex differences in vulnerability to GBH-induced neurotoxicity, oxidative stress, and dopaminergic dysfunction, as female mice may exhibit differential behavioral or biochemical outcomes. Both sexes might be included in future studies to have a better understanding of sex-dependent variation in GBH-induced neurotoxicity and the possible protective effect of TA.

Furthermore, this study acknowledges an absence of a TA-only group, an addition which would have allowed a better distinction between the inherent TA effects and its neuroprotective role against GBH-induced neurotoxicity. Although a previous report from a rotenone-induced rat model of PD has shown that TA administration in healthy rats resulted in no or minimal behavioral or oxidative alterations. Nonetheless, future investigations would incorporate a TA-only group to assist in delineating TAs’ baseline neurobehavioral effects. While this study demonstrates the neuroprotective actions of TA, its exact mechanism of action is not known, since TA is a high-molecular-weight polyphenol whose direct penetration across the blood–brain barrier remains incompletely characterized. However, TA’s action might have been indirect, through a systemic antioxidant effect, modulation of inflammatory pathways, or gut–brain axis interactions. Furthermore, the present study focused on dopamine because dopaminergic dyshomeostasis within the nigrostriatal pathway is an integral mechanism underlying toxin-induced motor dysfunction. While the concentrations of dopamine were measured, dopamine metabolites, including DOPAC and HVA, were not measured in the present study, which might have limited our interpretation of dopamine turnover dynamics. Our present focus does not in any way undermine the motor regulation role of other neurotransmitters, including acetylcholine, GABA, and glutamate. Future studies may explore whether chronic GBH exposure alters the dynamics of other neurotransmitters involved in motor control.

Conclusively, this study shows that TA significantly attenuated motor deficits in GBH-exposed mice and mitigated associated oxidative stress, neuro-inflammation, and dopaminergic neurotoxicity in mice. The results are suggestive of the neuroprotective potential of TA against environmental toxicant-induced neurotoxicity. Further studies are required to elucidate the precise mechanism of action of TA and determine whether this neuroprotective effect is relevant in neurodegenerative conditions involving dopaminergic dysfunction.

## Data Availability

The raw data supporting the conclusions of this article will be made available by the authors, without undue reservation.
